# Dietary Inflammatory Index and Type 2 Diabetes Mellitus in Adults: The Diabetes Mellitus Survey of Mexico City

**DOI:** 10.3390/nu10040385

**Published:** 2018-03-21

**Authors:** Edgar Denova-Gutiérrez, Paloma Muñoz-Aguirre, Nitin Shivappa, James R. Hébert, Lizbeth Tolentino-Mayo, Carolina Batis, Simón Barquera

**Affiliations:** 1 Nutrition and Health Research Center, National Institute of Public Health, Cuernavaca 62100, Mexico; edgar.denova@insp.mx (E.D.-G.); mltolentino@insp.mx (L.T.-M.); 2 Center for Research on Population Health , National Institute of Public Health, Cuernavaca 62100, Mexico; pmz.aguirre@gmail.com; 3 Cancer Prevention and Control Program, University of South Carolina, Columbia, SC 29208, USA; shivappa@email.sc.edu (N.S.); JHEBERT@mailbox.sc.edu (J.R.H.); 4 Department of Epidemiology and Biostatistics, Arnold School of Public Health, University of South Carolina, Columbia, SC 29208, USA; 5 Connecting Health Innovations LLC, Columbia, SC 29250, USA; 6 CONACYT-Nutrition and Health Research Center, National Institute of Public Health, Cuernavaca 62100, Mexico; Carolina.batis@insp.mx

**Keywords:** type 2 diabetes mellitus, dietary inflammatory index, obesity, Mexican population, survey

## Abstract

Diet and inflammation are both associated with type 2 diabetes mellitus (T2DM). In the present study, we aimed to assess the relation between the dietary inflammatory index (DII) and the presence of T2DM in Mexican adults participating in the Diabetes Mellitus Survey administered in Mexico City (DMS-MC). The study involved 1174 subjects (48.5% men) between 20–69 years of age. A validated semi-quantitative food frequency questionnaire was employed to evaluate dietary intake and to compute DII. The DII is based on scientific evidence about the association between dietary compounds and six established inflammatory biomarkers. Multivariate logistic regression models were used to estimate the odds ratios (ORs) and 95% confidence intervals (95% CIs) of DII in relation to T2DM. Our results suggest that subjects in the highest quintile of the DII had higher odds of T2DM (OR = 3.02; 95% CI: 1.39, 6.58; *p* = 0.005) compared to subjects in the lowest quintile of DII scores. Assessing possible effect modification, an association with T2DM was evident when comparing DII quintile 5 to quintile 1 for participants aged ≥ 55 years (OR = 9.77; 95% CI: 3.78, 25.50; *p* = 0.001). These results suggest that a pro-inflammatory diet is associated with significantly higher odds of T2DM among adult Mexicans.

## 1. Introduction

Low-grade, systemic inflammation, which provides the substrate for many chronic diseases, is characterized by elevated pro-inflammatory markers, including interleukin-6 (IL-6), and tumor necrosis factor-alpha (TNF-α) [1]. Chronic systemic inflammation has been linked to non-communicable diseases such as cancer, cardiovascular disease, and diabetes [2]. Globally, type 2 diabetes mellitus (T2DM) has become a serious public health problem, affecting 382 million people in 2013 [3] and had led to a loss of approximately 64 million disability adjusted life-years (DALYs) in 2015 [4]. Currently, 80% of people with T2DM live in low-income and middle-income countries [5]. In Mexico, T2DM prevalence has reached 14.7% of the population and two million DALYs lost in 2013 [5,6].

Recent evidence indicates that risk factors can induce chronic inflammation, which are also related with T2DM such as adiposity [7], a sedentary lifestyle [8], and diet [9]. In the past several decades, certain individual dietary factors have been widely investigated regarding their association with diabetes risk and inflammation, including high intake of saturated fatty acids, sugar-sweetened beverages, and starchy food, combined with low consumption of fruits, vegetables, and whole grains [10]. More recently, alternative approaches have been used in order to capture the complicated nutrient interactions and cumulative effects in the food matrix [11]. Based on evidence linking diet with inflammation and chronic conditions such as T2DM, we used a literature-derived, population-based dietary inflammatory index (DII) [12] to evaluate the potential inflammatory properties of diet. The DII has been validated with different inflammatory biomarkers [13,14]. Furthermore, it has been associated with components of metabolic syndrome [15], as well as a variety of chronic disease outcomes [16,17].

To the best of our knowledge, no study has yet explored the relation between the DII and T2DM in a Mexican population. Thus, we aimed to explore the association between DII and the prevalence of T2DM in adults participating in the Diabetes Mellitus Survey in Mexico City 2015 (DMS-MC 2015).

## 2. Materials and Methods

### 2.1. Study Design

The present analysis was conducted with data from the DMS-MC 2015, a cross-sectional probabilistic population-based survey, designed and implemented by our group at the National Institute of Public Health (INSP by its Spanish acronym), representative of adults aged 20–69 years living in Mexico City. The DMS-MC 2015 sample was randomly stratified into clusters according to city district. In the first stage, 16 primary sampling units were selected and by means of a probabilistic and systematic sampling, six secondary sampling units were designated. Then, six houses per secondary sampling unit were included. Finally, per household, we evaluated up to two adults between 20–69 years of age. Fasting venous blood samples were collected in a subsample of participants randomly selected based on estimations of the Mexican National Health and Nutrition Survey 2012 [18].

For the present analysis, we excluded participants with >10% blank items on their food frequency questionnaires, and who did not consume between 600 kcal and 5500 kcal daily (*n* = 10), determined with the standard deviation method suggested by Rosner [19]. Additionally, we excluded participants with incomplete biomarkers data or with missing information on other important covariates (*n* = 142). Finally, we excluded those subjects with more than 12 months past their T2DM diagnosis date (*n* = 90). A total of 1174 individuals were included in our final sample.

This study was managed according to the Declaration of Helsinki guidelines. The Research, Ethics and Biosecurity Committee at INSP reviewed and approved the study protocol (No. 1658) and informed consent forms (No. B04). Written informed consent was obtained from each participant.

### 2.2. Dietary Assessment

To assess dietary intake, a previously validated semi-quantitative food frequency questionnaire (SFFQ) [20] was used. The instrument describes the consumption of 140 foods over the past seven days prior to the interview. For each food, a commonly used portion size was specified on the SFFQ. Frequency of food consumption was characterized by set categories ranging from never to six. First, frequency was expressed as times per day, but then was converted into portion size per day. To compute the energy (kcal/day) and daily nutrient intake, we multiplied the frequency of consumption of each food by the estimated nutrient content with a comprehensive database of food contents, compiled by the INSP [21]. The SFFQ was administered by interviewers and were collected by personnel trained using standardized data collection and entry procedures.

### 2.3. Dietary Inflammatory Index (DII) Assessment

SFFQ-derived dietary data was used to calculate DII scores for each participant. A complete description of the DII is available elsewhere [12]. Briefly, the body of literature on DII consists of all qualifying publications between 1950 and 2010 reporting one or more associations between dietary components and the following inflammatory markers: IL-1β, IL-4, IL-6, IL-10, TNF-α and C-reactive protein [12]. A total of 45 different food parameters were identified as being related to the six inflammatory biomarkers in the literature review. Each was assigned a “food parameter-specific inflammatory effect score” through a process of counting the number of studies reporting a pro-inflammatory, anti-inflammatory, and no inflammatory effect on one or more of the six inflammatory markers, and weighing the scores by study design and size of the literature for each food parameter/inflammatory marker relation. In previous analyses, the DII was positively correlated with circulating level of high-sensitivity C-reactive protein (hs-CRP) [13,22].

To calculate DII scores for the participants of this study, the dietary data was first linked to the world database that provided estimates of a mean intake and standard deviation for each food parameter [12]. These then became the multipliers to express an individual’s exposure relative to the “standard global mean” as a *z*-score. This was achieved by subtracting the “standard global mean” from the amount reported and dividing this value by the standard deviation. Since data was skewed to the right (a common occurrence with dietary data), we converted this value into a centered percentile score. The centered percentile score from every individual was multiplied by the food parameter-specific inflammatory effect score in order to obtain a food parameter-specific DII score for an individual. All of the food parameter-specific DII scores were then summed to create the overall DII score for every participant in the study [12]. DII scores for individuals in the DMS-MC 2015 were calculated using the 27 food items and nutrients (out of the 45 possible items) for which we had intake data available from the SFFQ: carbohydrate, protein, fat, alcohol, fiber, cholesterol, saturated fatty acids, mono-unsaturated fatty acids, poly-unsaturated fatty acids, omega 3 fat, omega 6 fat, trans fat, niacin, thiamin, riboflavin, vitamin B12, vitamin B6, iron, magnesium, zinc, vitamin A, vitamin C, vitamin E, folic acid, beta carotene, garlic, and onion. Since the DII was calculated per 1000 calories of food consumed we used the energy-standardized version of the world database to control for the effect of total energy intake.

### 2.4. Biomarkers Assessment

A fasting venous blood sample (fasting time was ≥8 h) from an antecubital vein was collected from each participant. Serum aliquots were stored in cryovials and transported to a laboratory, where the aliquots were stored at −70 °C until they were used for analysis.

Plasma triglycerides were measured with a colorimetric method following enzymatic hydrolysis performed with the lipase technique. Total cholesterol, high-density lipoprotein-cholesterol (HDL-c), and low-density lipoprotein-cholesterol (LDL-c) were measured using the colorimetric method following enzymatic assay. Additionally, plasma glucose was measured with the enzymatic colorimetric methods by using glucose oxidize. Finally, the proportion of hemoglobin A1c (HbA1c) was determined using the immunocolorimetric method [23].

### 2.5. Type 2 Diabetes Mellitus Definition

For the present study, subjects who declared to have a previous T2DM physician-diagnosis independent of their survey glucose concentration were called “previously diagnosed”. Of these, respondents who were diagnosed with T2DM less than 11 months prior to administration of the SFFQ, had fasting glucose concentrations ≥126 mg/dL (at the moment of the survey) and poor glycemic control (HbA1c (%) ≥ 6.5) [24]. These subjects were considered participants with T2DM. Furthermore, subjects whose glucose concentration in the fasting blood sample taken during the survey was ≥ 126 mg/dL and had levels of (HbA1c (%) ≥ 6.5) were defined as displaying fasting glucose and/or HbA1c values consistent with T2DM diagnostic criteria and were also defined as participants with T2DM.

### 2.6. Anthropometric and Blood Pressure Assessment

Participants’ height and weight were measured by trained personal using standardized procedures. Height was measured by using a conventional stadiometer (SECA 213, Medical Measuring Systems and Scales, Hamburg, Germany) to the nearest 0.1 cm and body weight was measured with a previously calibrated electronic (SECA 874, Medical Measuring Systems and Scales, Hamburg, Germany) scale with a precision of 0.1 kg. Body mass index (BMI = weight (kg)/height (m^2^)) was calculated based on measured weight and height. We defined overweight/obesity as BMI ≥25 kg/m^2^. Waist circumference was measured to the nearest 0.1 cm at the high point of the iliac crest at the end of normal expiration, with a measuring tape, which was placed below any clothing, directly touching the participant’s skin. Abdominal obesity was defined as a waist circumference of ≥90 cm in men and ≥80 cm in women [25].

Subjects’ blood pressure was measured twice by a trained personal using an automatic medical grade monitor (OMROM HEM-907, OMROM Mexico, Mexico City, Mexico). The first measurement was taken after five minutes of rest, while participants were sitting with the dominant arm supported at heart level. The second measurement was taken in the same way, five minutes after the first.

### 2.7. Physical Activity Assessment

Physical activity was evaluated with a previously used and validated [26] short version of the international physical activity questionnaire (s-IPAQ). The questionnaire includes 9 items that assesses time spent performing moderate-intense physical activity for at least 10 min for each activity over seven days. The s-IPAQ data was analyzed in agreement with IPAQ protocol [27], as follows: first, physical activity interval duration gathered in hours was converted into minutes; second, data which was described as a weekly frequency was transformed into an average daily time; and third, subjects whose responses were “do not know”, or “refused”, or had “missing data” for time duration or frequency were removed from the present analysis. Based on the reported time spent performing moderate to intense physical activity, participants were classified as inactive (<150 min/week), physically active (150–299 min/week), or highly active (≥300 min/week) according to the World Health Organization (WHO) physical activity guidelines [28].

### 2.8. Socioeconomic Status and Education Assessment

Socioeconomic status (SES) was constructed by combining eight variables that assessed household characteristics, goods, and available services including: construction materials of the floor, ceiling, and walls; household goods (stove, microwave, washing machine, refrigerator and boiler); and electrical goods (television, computer, radio and telephone). The index was divided into tertiles and used as a proxy for low, medium, and high SES. Education level was stratified into three groups according to the highest level of education obtained: primary or less, secondary/high school, and/or higher education.

### 2.9. Other Participant Characteristics

Participants completed two self-administered questionnaires (home and individual level) and delivered detailed information regarding their demographic characteristics (e.g., age, sex, education, marital status), self-perception of body weight, past medical history, current medication use, lifestyle information (e.g., diet, physical activity, smoking status, alcohol consumption, etc.), depression symptoms, sleep quantity, and information on reproductive history (for females).

### 2.10. Statistical Analysis

We conducted descriptive analyses of the main characteristics of interest to assess adherence to model assumptions. One-way analysis of variance (ANOVA) was used to test for differences for general characteristics across quintiles of DII, while, chi-square tests were used to evaluate the distribution of qualitative variables across DII quintiles. To evaluate the magnitude of the association between specific DII and diabetes, we estimated multivariable adjusted odds ratios (OR) 95% CI using logistic regression models. In all multivariate models, the first quintile of the DII score was considered the reference. The Mantel–Haenszel extension chi-square test was used to assess the overall trend of OR across increasing quintile of DII scores.

Additionally, to assess possible effect modification, we conducted a stratified analysis by age groups (<55 years vs. ≥55 years), sex, BMI (<25.0 kg/m^2^ vs. ≥25.0 kg/m^2^), and physical activity (inactive vs. active or highly active). We tested the significance using a likelihood ratio test by comparing a model with the main effects of each intake and the stratified variable and the reduced model interaction terms with only the main effects.

All *p*-values presented are two-tailed, *p* < 0.05 was considered significant. All analyses were performed using STATA software (College Station, StataCorp LP, TX, USA), version 13.0.

## 3. Results

Participants’ baseline characteristics are shown in Table 1. A total of 1174 subjects (48.5% men) between 20–69 years were included in the present analysis. The overall prevalence of T2DM was 13.6%; of these, 10.1% were previously diagnosed individuals, whereas 3.5% were individuals with glucose and/or HbA1c values consistent with T2DM definition at the time of the survey. The mean age among participants with T2DM was 52.3 years. Subjects with T2DM had a significantly higher prevalence of obesity, abdominal obesity, had lower levels of physical activity and intake of cereal fiber, and also, as expected, levels of total cholesterol, triglycerides, and glycated hemoglobin were all significantly higher in participants with T2DM.

According to DII score quintiles, subjects with the most pro-inflammatory diet were significantly older, less educated, had lower SES, smoked less, had a higher prevalence of obesity, abdominal obesity, and T2DM (Table 2). The most pro-inflammatory diet was characterized by a higher consumption of carbohydrates (56.1%; (95% CI: 54.3, 57.8) vs. 53.8% (52.6, 55.1)), red meat and processed meat (78.3 g/day (66.3, 90.2) vs. 40.1 g/day (33.7, 46.5)), refined cereals (173.9 g/day (147.1, 200.6) vs. 109.2 g/day (96.0, 122.5)), and soft drinks (401.7 mL/day (343.3, 460.1) vs. 69.5 mL/day (51.6, 87.4)) compared to the most anti-inflammatory diet. All of these differences were statistically significant. Also, significantly decreasing trends were observed for the anti-inflammatory nutrients: fiber, vitamin C, vitamin D, and magnesium (Table 3).

After adjusting for age and sex, the odds of having T2DM, across all DII score quintiles were 1.00, 1.63, 1.80, 1.84, and 2.29 (95% CI: 1.11, 4.75; *p* = 0.01). Finally, after additional adjustment for co-variables, we observed that subjects with the most pro-inflammatory diet had approximately three times greater odds of having T2DM (OR: 3.02, 95% CI: 1.39, 6.58; *p* = 0.005), compared to individuals in the lowest DII quintile (Table 4).

Additionally, a sensitivity analysis included individuals with glucose and/or HbA1c values consistent with T2DM definition at the time of the survey. In this case, we observed that subjects with the most pro-inflammatory diet had greater odds of having T2DM (OR = 3.56; 95% CI: 1.13, 9.11) (data not shown).

**Figure 1:**
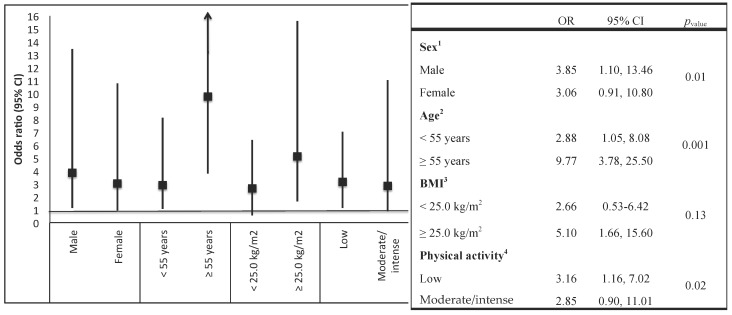
Subgroup analysis. Odds ratios (95% CI) for the association between extreme quintiles of the dietary inflammatory index (DII) and type 2 diabetes mellitus (T2DM). ^1^ Adjusted for age (years), physical activity (inactive vs. active/highly active), hours of television watching; tobacco use (current, past, and never), socioeconomic status (low, medium, and high), education (elementary and secondary education, high school, and Bachelor’s degree or higher), family history of diabetes mellitus (yes vs. no), personal history of hypertension (yes vs. no), medication use (yes vs. no), multivitamin use (yes vs. no), alcohol intake (gr/day), body mass index (<25.0 vs. ≥25.0 kg/m^2^). ^2^ Adjusted for sex, physical activity (inactive vs. active/highly active), hours of television watching; tobacco use (current, past, and never), socioeconomic status (low, medium, and high); education (elementary and secondary education, high school, and Bachelor’s degree or higher), family history of diabetes mellitus (yes vs. no), personal history of hypertension (yes vs. no), medication use (yes vs. no), multivitamin use (yes vs. no), alcohol intake (gr/day), body mass index (<25.0 vs. ≥25.0 kg/m^2^). ^3^ Adjusted for age (years), sex, physical activity (inactive vs. active/highly active), hours of television watching, tobacco use (current, past, and never), socioeconomic status (low, medium, and high); education (elementary and secondary education, high school, and Bachelor’s degree or higher), family history of diabetes mellitus (yes vs. no), personal history of hypertension (yes vs. no), medication use (yes vs. no), multivitamin use (yes vs. no), alcohol intake (gr/day). ^4^Adjusted for age (years), sex, hours of television watching, tobacco use (current, past, and never); socioeconomic status (low, medium, and high); education (elementary and secondary education, high school, and Bachelor’s degree or higher), family history of diabetes mellitus (yes vs. no), personal history of hypertension (yes vs. no), medication use (yes vs. no), multivitamin use (yes vs. no), alcohol intake (gr/day), body mass index (<25.0 vs. ≥25.0 kg/m^2^).

In addition, we evaluated the effect-modifying role of age; we observed a greater association with T2DM contrasting DII extreme quintiles (Q_5_ vs. Q_1_) for participants aged ≥ 55 years (OR = 9.77; 95% CI: 3.78, 25.50; *p* = 0.001) (Figure 1).

## 4. Discussion

In the Mexico City Diabetes Mellitus Survey, we observed that participants in the DII score highest quintile, representing the most pro-inflammatory diet, had higher odds of T2DM (independent of other diabetes risk factors) compared with participants in the DII score lowest quintile (maximum anti-inflammatory potential). When we stratified by age (<55 years vs. ≥55 years), we observed positive associations between DII and T2DM, with larger magnitude of association among participants ≥55 years of age.

In the present study, we used a previously derived and validated DII score [13] to appraise the capacity of a pro-inflammatory diet on T2DM. The DII scores computed from a Mexican population included 27 food parameters, ranged from −5.49 to +4.12 with a mean of −0.68. This result is consistent with prior publications conducted in Italy, France, and Spain [16,17,29,30]. For example, Ramallal and colleagues at the University of Navarra cohort study reported a DII score ranging from −5.14 to +3.97 [30].

We also evaluated associations between food groups and nutrient intake and the DII, and found that subjects in the highest DII quintile (most pro-inflammatory diet) consumed more red and processed meat, eggs, refined cereals, and soft drinks, and had a lower intakes of vegetables, fruits, fish, and seafood. Similar to these results, the “PREvención con DIeta MEDiterránea” (PREDIMED) study found that consumption of vegetables and fruit was less frequent among men and women in the highest DII quintile [29].

Regarding nutrient intake, we observed that subjects with the most pro-inflammatory diet had lower intakes of polyunsaturated fatty acids, fiber, magnesium, and some vitamins. Similar to our results, the PREDIMED study found that subjects in the highest DII quintile consumed fewer polyunsaturated fatty acids, vitamins, and fiber. Moreover, other studies using the dietary pattern or dietary score approach, have observed an inverse association between healthy diets or patterns (mainly characterized by fruits, vegetables, whole grains and fiber) and inflammation and T2DM, as well as a positive association with Western patterns or unhealthy diet scores [9,10,30,31].

In the present analysis, we studied the association between DII and T2DM. We observed that subjects in the highest DII quintile had approximately three times greater odds of T2DM compared with subjects in the lowest DII quintile. The findings in our analysis agree with prior studies that evaluate the relation between diet and T2DM. For example, in the Insulin Resistance Atherosclerosis Cohort study, including subjects with a diet high in red meat, low-fiber cereals, fried potatoes, eggs, cheese, and low in wine had approximately 4.5 times greater risk of T2DM comparing extreme quartiles of the dietary pattern [31]. Similar results were observed in the Nurses’ Health Study [32], where a pattern higher in sugar-sweetened soft drinks, refined grains, and processed meat but low in vegetables was associated with an increased risk of T2DM (OR = 3.09; 95% CI: 1.99, 4.79; compared to diet characterized by lower consumption of these dietary components). On the other hand, multiple prospective studies have reported an inverse association between the adherence to Mediterranean diet (with anti-inflammatory effects) [33] and the risk of T2DM [34]. Furthermore, a recent study [35] that evaluated the relation between DII and the risk of prediabetes found that subjects in the highest DII tertile had higher odds of prediabetes (OR = 18.88; 95% CI: 7.02, 50.82) compared to those who consumed a more anti-inflammatory diet. Additionally, most of the highly consumed food groups in the most pro-inflammatory DII quintile (red and processed meat, refined cereals, and soft drinks) have been associated with T2DM and with inflammatory markers [10]. For example, soft drinks have been linked to T2DM and inflammatory biomarkers due to their significant contribution to the glycemic load [36]. Whereas, low intakes of whole grains, fruits, and vegetables in the highest DII quintile have been related with reduced diabetes risk, probably mediated by a reduction of CRP, and certain interleukins, and by an improvement in the endothelial function [37].

Other results in the present study indicate that a pro-inflammatory effect of diet on T2DM could be particularly unfavorable among older (≥55 years) or inactive individuals (<150 min/week). Although not statistically significant, there was a suggestion of an interaction between DII and overweight/obesity in relation to T2DM. Despite the lack of significance, this result could be biologically important. Similar results were obtained in the Multiethnic Cohort (MEC) Study. In this study, the stratified analysis suggests that, among men, the risk for T2DM increased with higher consumption of fat and meat patterns among overweight (Hazard ratio = 1.49; 95% CI: 1.23, 1.81) and obese (HR = 1.57; 95% CI: 1.16, 2.12) individuals [38]. In women, similar results were observed, although these results were not statistically significant. In our case, this finding should be interpreted with caution; it is difficult to determine, with our study design, the role of BMI in the causal sequence (i.e., whether it is a confounder or an effect modifier). The relation between DII and T2DM remained strong after adjustment for BMI, indicating that the DII may be associated with diabetes in all individuals, but especially among obese subjects.

The connection between DII and T2DM could be explained through the effect of a pro-inflammatory diet on insulin resistance that has been linked to the inflammatory process [39]. In this sense, prior studies suggest a positive relationship between markers of chronic inflammation, including CRP, IL-1β, IL-6, and TNF-α, and insulin resistance [39]. Furthermore, it has been established that insulin resistance increases levels of these adhesion molecules in diabetic and nondiabetics individuals [40]. On the other hand, the functional effects of food or food groups and nutrients like meat, processed meat, refined cereals, and soft drinks, which corresponded with high DII scores in our study, have been also shown to influence systemic inflammation [32].

Strengths of our study include: (1) the random stratified cluster design of the DMS-MC; (2) the use of previously validated questionnaires; (3) the inclusion of many potential demographic, behavioral, and other factors as potential effect modifiers or confounders in the multivariate analysis; and (4) the use of a validated DII score specially constructed to assess the inflammatory potential of any diet [12,13]. As reported in other studies [17,29], the DII can be adapted for use in different populations including the Mexican population providing results that can be compared to those from studies based in diverse populations in many parts of the world.

Some potential weaknesses of our study need to be highlighted. Because of its cross-sectional design, this study cannot infer causality. Therefore, these results need to be further investigated in future longitudinal studies. Although the SFFQ used in this study had been previously validated [20], other possible limitations are related to information bias. In this regard, it is important to note that dietary factors used to calculate the DII were evaluated from a single measurement of SFFQ, which is subject to random error that would tend to underestimate the true association between DII and T2DM in our study. As observed in previous studies [29], some dietary components such as saffron, thyme, turmeric, and others were not available to compute the DII in the present analysis; however, as they are not habitually consumed in large quantities or common in Mexican diet, they may not have an important impact on the DII score. Another probable weakness could be related to the T2DM definition; nevertheless, we provided appropriate allowance for the prevalent ascertainment in the present analysis. In other words, prevalent cases of T2DM (previously diagnosed) were included only if they were recently diagnosed (less than 11 months), had fasting glucose concentrations ≥126 mg/dL, and had poor glycemic control (HbA1c ≥ 6.5) at the time of the survey. Additionally, we conducted a sensitivity analysis including only subjects diagnosed at the time of the survey and we observed similar results (OR = 3.56; 95% CI: 1.13, 9.11). We adjusted for many potentially confounding factors, however, residual confounding due to measurement error, mainly in assessing some self-reported lifestyle variables or due to insufficient control in statistical models, might have produced some degree of bias in our results. However, it is improbable that such a bias would explain the consistently robust association observed between DII and T2DM.

## 5. Conclusions

Our data suggest that a higher DII score (revealing a more pro-inflammatory diet) was associated with increased odds of T2DM compared with DII scores in the lowest quintile of the DII (indicative of an anti-inflammatory diet) among participants from the DMS-MC. In addition, we observed that the magnitude of the association appeared to be more pronounced among overweight/obesity subjects, older individuals, and those with low levels of physical activity. Further longitudinal investigations evaluating this relationship are needed to determine causality. Finally, the DII may be an important tool to characterize the diet of the Mexican population and further explore associations with non-communicable diseases.

## Figures and Tables

**Table 1:** Characteristics of the study population: The Diabetes Mellitus Survey of Mexico City, 2015.

Variables	Overall Study	Non-T2DM Subjects	T2DM Subjects	*p*-Value ^a^
	(*n* = 1174)	(*n* = 973)	(*n* = 201)	
**Sex, %**				
Men	48.5	48.4	48.7	0.17
Women	51.5	51.6	51.3	
Age (years) ^b^	39.9 (0.48)	38.0 (0.46)	52.3 (0.83)	<0.001
**Socioeconomic status, %**				
Low	21.9	20.2	33.5	<0.001
Medium	36.4	36.1	37.4	
High	41.7	43.7	29.1	
**Education, %**				
Elementary and secondary education	19.3	16.5	37.4	<0.001
High school	27.2	26.5	32.0	
Bachelor’s degree or higher	53.5	57.0	30.6	
**Smoking status, %**				
Current	45.6	45.2	48.8	0.41
Past	11.6	12.1	7.8	
Never	42.8	42.7	43.4	
**Physical activity, %**				
Inactive	23.0	22.8	24.4	0.17
Active/highly active	77.0	77.2	75.6	
**Family history of DM2, %**	41.9	38.2	66.5	<0.001
**Hypertension, (%)**	15.9	11.5	44.1	<0.001
Body mass index (kg/m^2^) ^b^	28.8 (0.22)	28.2 (0.23)	31.2 (0.48)	<0.001
**Body mass index, %**				
Normal (<25.0 kg/m^2^)	25.5	28.2	9.1	<0.001
Overweight (≥25.0 to <30.0 kg/m^2^)	40.2	39.9	41.8	
Obesity (≥30.0 kg/m^2^)	34.3	31.9	49.1	
**Abdominal obesity, %**	43.1	38.9	70.2	<0.001
Glucose (mg/dL) ^b^	107.7 (2.0)	91.3 (0.53)	213.8 (7.7)	<0.001
Glycated hemoglobin (HbA1c %) ^b^	5.9 (0.07)	5.3 (0.02)	9.8 (0.23)	<0.001
Triglycerides (mg/dL) ^b^	205.6 (6.3)	189.8 (6.3)	307.3 (15.1)	<0.001
Total cholesterol (mg/dL) ^b^	189.1 (1.5)	186.2 (1.5)	207.8 (4.0)	<0.001
High density lipoprotein (mg/dL) ^b^	42.3 (0.41)	42.8 (0.49)	39.9 (0.68)	0.39
Low density lipoprotein (mg/dL) ^b^	84.0 (2.1)	80.9 (2.0)	87.6 (4.9)	<0.001
**Dietary variables**				
Energy intake (kcal/day) ^c^	2224 (2106–2342)	2329 (2196–2462)	1801 (1673–1927)	<0.001
Carbohydrates (% energy) ^c^	55.4 (54.7–56.1)	55.1 (54.3–55.9)	56.8 (55.4–58.1)	0.06
Total fats (% energy) ^c^	31.1 (30.5–31.7)	31.3 (30.6–31.9)	30.2 (29.3–31.2)	0.03
Saturated fats (% energy) ^c^	11.6 (11.3–11.9)	11.8 (11.5–12.0)	11.2 (10.7–11.6)	0.001
Monounsaturated fatty acids (% energy) ^c^	10.9 (10.7–11.1)	11.0 (10.7–11.2)	10.4 (10.0–10.8)	0.03
Polyunsaturated fatty acids (% energy) ^c^	7.0 (6.8–7.2)	7.0 (6.8–7.2)	7.0 (6.8–7.3)	0.61
Fiber (g/day) ^c^	27.7 (25.9–29.6)	28.3 (26.3–30.3)	25.6 (23.8–27.4)	<0.001
Alcohol intake (g/day) ^c^	9.4 (7.4–11.3)	10.4 (8.2–12.6)	5.0 (2.5–7.5)	0.003
Magnesium (mg/day) ^c^	409.7 (386.5–432.9)	417.7 (392.3–443.1)	377.3 (351.4–403.2)	<0.001

^a^ *p* <0.05, difference in mean and proportion between diabetic and non-diabetic subjects. Values were determined using a student’s *t*-test for continuous variables and Chi-square test for categorical variables. ^b^ Mean and (standard error); ^c^ Mean and 95% confidence intervals (95% CI). T2DM, type 2 diabetes mellitus; HbA1c, hemoglobin A1c.

**Table 2:** Characteristics of participants according to quintiles of the dietary inflammatory index: The Diabetes Mellitus Survey of Mexico City, 2015.

	Dietary Inflammatory Index					
	Quintile 1: Most Anti-Inflammatory	Quintile 2	Quintile 3	Quintile 4	Quintile 5: Most Pro-Inflammatory	*p*-Value ^a^
	(*n* = 235)	(*n* = 235)	(*n* = 235)	(*n* = 235)	(*n* = 234)	
Mean DII-density	−3.05	−1.85	−0.81	0.24	1.79	<0.001
**Sex, %**						
Men	24.7	37.5	38.9	40.1	48.9	<0.001
Women	75.3	62.5	61.1	59.9	51.1	
Age (years) ^b^	39.9 (0.91)	42.9 (1.2)	45.5 (1.11)	46.2 (1.08)	48.5 (1.05)	0.001
**Socioeconomic status, %**						
Low	21.5	22.8	21.5	22.3	34.8	<0.001
Medium	32.2	39.0	38.5	41.0	34.3	
High	46.3	38.2	40.0	36.7	30.9	
**Education, %**						
Elementary and secondary education	10.6	26.1	27.3	27.6	34.4	<0.001
High school	24.5	21.3	27.2	29.8	31.7	
Bachelor’s degree or higher	59.9	52.6	45.5	42.6	33.9	
**Smoking status, %**						
Current	52.9	51.8	47.8	39.5	31.7	<0.001
Past	10.5	6.1	10.0	17.9	15.2	
Never	36.6	42.1	42.2	42.6	53.1	
**Physical activity, %**						
Inactive	25.7	27.8	29.5	26.5	23.8	0.14
Active/highly active	74.3	72.2	70.5	73.5	76.2	
Body mass index (kg/m^2^) ^b^	28.1 (0.38)	28.3 (0.40)	28.7 (0.47)	28.9 (0.45)	29.8 (0.48)	0.007
**Body mass index, %**						
Normal (<25.0 kg/m^2^)	29.2	25.3	23.5	25.4	20.8	0.005
Overweight (≥25.0 to <30.0 kg/m^2^)	38.5	38.8	41.4	38.6	36.2	
Obesity (≥30.0 kg/m^2^)	32.3	35.8	35.1	36.0	43.0	
**Abdominal obesity, %**						
Yes	39.3	47.2	48.3	50.3	54.2	<0.001
Glucose (mg/dL) ^b^	100.9 (3.6)	103.8 (2.8)	108.8 (3.6)	112.4 (3.9)	117.2 (4.7)	<0.001
Glycated hemoglobin (HbA1c %) ^b^	5.7 (0.10)	5.8 (0.12)	6.2 (0.14)	6.2 (0.13)	6.7 (0.11)	0.006
Triglycerides (mg/dL) ^b^	200.6 (10.8)	188.3 (12.3)	217.9 (13.0)	203.1 (11.6)	216.7 (13.1)	0.96
Total cholesterol (mg/dL) ^b^	187.7 (3.1)	181.6 (3.0)	192.4 (3.5)	190.9 (3.2)	196.4 (3.3)	0.02
High density lipoprotein (mg/dL) ^b^	42.8 (1.03)	41.2 (0.81)	42.1 (0.75)	42.1 (0.76)	41.7 (0.71)	0.17
Low density lipoprotein (mg/dL) ^b^	81.2 (4.4)	85.2 (3.4)	80.2 (4.7)	88.7 (4.4)	88.9 (4.8)	0.79
**Type 2 Diabetes Mellitus, %**						
Yes	6.4	11.1	13.3	16.2	22.8	<0.001

^a^ *p*-values were determined using analysis of variance (ANOVA) test for continuous variables and Chi-square test for categorical variables. ^b^ Data are given as means, with standard error (SE) in parentheses, unless otherwise specified.

**Table 3:** Nutrient and food consumption according to quintiles of the dietary inflammatory index: in the Diabetes Mellitus Survey of Mexico City, 2015.

Variables	Dietary Inflammatory Index										*p*-Value*^a^*
	Quintile 1: Most Anti-Inflammatory		Quintile 2		Quintile 3		Quintile 4		Quintile 5: Most Pro-Inflammatory		
	Mean	(95% CI)	Mean	(95% CI)	Mean	(95% CI)	Mean	(95% CI)	Mean	(95% CI)	
Carbohydrates intake (% energy) ^b^	53.8	(52.6, 55.1)	55.0	(53.7, 56.4)	56.0	(55.2, 58.4)	56.1	(54.7, 57.5)	56.1	(54.3, 57.8)	<0.001
Protein intake (% energy)	14.4	(13.9, 14.8)	13.9	(13.4, 14.6)	13.7	(13.1, 14.0)	13.0	(12.6, 13.5)	12.6	(11.7, 13.7)	<0.001
Total fat intake (% energy)	31.8	(30.9, 32.9)	30.8	(29.8, 31.6)	30.3	(29.2, 31.3)	31.2	(30.1, 32.3)	31.3	(29.9, 32.7)	0.08
Saturated fats (% energy)	11.4	(10.9, 11.9)	11.7	(11.2, 12.2)	11.3	(10.9, 11.8)	11.3	(10.8, 11.8)	12.3	(11.5, 13.2)	0.51
Monounsaturated fats (% energy)	11.2	(10.8, 11.6)	10.8	(10.4, 11.2)	10.6	(10.1, 11.1)	10.9	(10.4, 11.4)	10.9	(10.3, 11.4)	0.20
Polyunsaturated fats (% energy)	7.6	(7.3, 7.9)	7.2	(6.9, 7.5)	6.9	(6.6, 7.2)	6.8	(6.5, 7.1)	6.7	(6.5, 7.0)	<0.001
Fiber (g/day)	42.7	(40.0, 45.4)	31.5	(27.8, 35.2)	24.7	(23.5, 25.9)	24.2	(22.6, 25.9)	13.3	(12.7, 14.0)	<0.001
Alcohol consumption (g/day)	15.2	(9.4, 21.0)	11.8	(7.2, 16.4)	6.6	(2.2, 11.0)	6.6	(1.2, 13.2)	2.5	(1.2, 3.9)	<0.001
Magnesium (mg/day)	600.3	(568.8, 631.8)	453.5	(402.1, 504.8)	365.5	(350.3, 380.7)	355.9	(333.7, 388.2)	219.5	(208.6, 230.5)	<0.001
Vitamin C (mg/day)	374.4	(344.5, 404.2)	288.0	(258.2, 317.8)	194.5	(181.0, 208.2)	160.6	(141.4, 179.7)	82.0	(72.9, 91.2)	<0.001
Vitamin A (μg/day)	1900.2	(1666.1, 2134.2)	1219.9	(1071.6, 1368.2)	830.1	(772.7, 887.6)	701.5	(635.1, 767.9)	396.3	(360.0, 432.6)	<0.001
Vitamin E (mg/day)	13.0	(12.3, 13.6)	8.9	(7.6, 10.1)	6.5	(6.2, 6.8)	6.5	(6.2, 7.3)	3.4	(3.4, 3.9)	<0.001
Vitamin D (μg/day)	7.7	(7.1, 8.3)	5.1	(4.4, 5.7)	4.4	(3.9, 4.9)	3.8	(3.4, 4.2)	2.5	(2.5, 3.1)	<0.001
Vegetables (g/day)	370.0	(321.2, 418.8)	245.5	(212.8, 278.4)	139.4	(121.3, 157.5)	112.2	(95.9, 128.5)	69.3	(60.0, 78.6)	<0.001
Fruits (g/day)	327.6	(293.3, 362.0)	259.9	(225.9, 294.0)	178.8	(162.2, 195.5)	134.5	(114.3, 154.6)	74.2	(64.4, 84.0)	<0.001
Legumes (g/day)	67.4	(49.5, 85.3)	51.0	(29.2, 72.6)	31.6	(26.0, 37.2)	33.7	(26.6, 40.8)	21.2	(16.2, 26.1)	<0.001
Fish and seafood (g/day)	24.1	(15.0, 33.1)	13.9	(4.7, 23.0)	10.8	(5.9, 15.7)	10.4	(5.1, 15.7)	3.2	(1.5, 4.9)	<0.001
Dairy products (g/day)	242.5	(209.9, 275.1)	225.8	(188.9, 262.6)	177.9	(142.3, 213.5)	172.5	(134.3, 210.8)	139.2	(109.3, 169.1)	<0.001
Red meat and processed meat (g/day)	40.1	(33.7, 46.5)	46.9	(41.3, 52.6)	55.0	(44.4, 65.5)	55.3	(41.8, 68.7)	78.3	(66.3, 90.2)	<0.001
Eggs (g/day)	28.9	(23.8, 33.9)	30.8	(25.1, 36.5)	32.2	(25.2, 39.2)	35.5	(28.4, 42.6)	34.3	(26.6, 41.9)	<0.001
Refined cereals (g/day)	109.2	(96.0, 122.5)	145.0	(129.8, 160.3)	145.4	(125.5, 165.3)	158.2	(122.7, 193.6)	173.9	(147.1, 200.6)	<0.001
Potatoes (g/day)	9.7	(5.2, 14.2)	11.8	(7.7, 15.8)	11.5	(7.8, 15.3)	8.3	(4.9, 11.7)	7.1	(5.1, 9.2)	0.87
Soft drinks (mL/day)	69.5	(51.6, 87.4)	146.9	(109.6, 184.1)	219.5	(175.7, 263.3)	262.4	(202.2, 322.6)	401.7	(343.3, 460.1)	<0.001

^a^ *p*-values were determined using ANOVA test. ^b^ Data are given as means, with 95% CI in parentheses.

**Table 4:** Odds ratio (OR) and 95% confidence intervals (CI) for the relation between the dietary inflammatory index and T2DM in the Diabetes Mellitus Survey of Mexico City, 2015.

	Dietary Inflammatory Index									*p*-Value
	Quintile 1: Most Anti-Inflammatory	Quintile 2		Quintile 3			Quintile 4	Quintile 5: Most Pro-Inflammatory		
	OR	OR	(95% CI)	OR	(95% CI)	OR	(95% CI)	OR	(95% CI)	
Model I	1.0	1.55	(0.86, 2.78)	1.80	(0.90, 3.56)	1.78	(0.93, 3.53)	2.29	(1.11, 4.75)	0.01
Model II	1.0	1.73	(0.94, 3.20)	1.88	(0.91, 3.89)	1.97	(1.06, 3.64)	3.00	(1.38, 6.55)	0.005
Model III	1.0	1.80	(0.95, 3.38)	2.01	(0.97, 4.13)	2.10	(1.07, 3.78)	3.02	(1.39, 6.58)	0.005

Model I: Adjusted by age and sex. Model II: Model I plus physical activity (inactive vs. active/highly active); hours of television watching; tobacco use (current, past, and never); socioeconomic status (low, medium, and high); education (elementary and secondary education, high school, and Bachelor’s degree or higher); family history of diabetes mellitus (yes vs. no); persona history of hypertension (yes vs. no), medication use (yes vs. no), multivitamin use (yes vs. no), and alcohol intake (gr/day). Model III: Model II plus body mass index (<25.0 vs. ≥25.0 kg/m^2^).

## Supplementary Materials

Supplementary File 1
