# Supplementary material for: Dietary Inflammatory Index and Type 2 Diabetes Mellitus in Adults: The Diabetes Mellitus Survey of Mexico City
Source: Nutrients. 2018 Mar 21;10(4):385. doi: 10.3390/nu10040385 (PMC5946170; doi:10.3390/nu10040385)
Supplement: Supplementary File 1 [file nutrients-10-00385-s001.pdf]

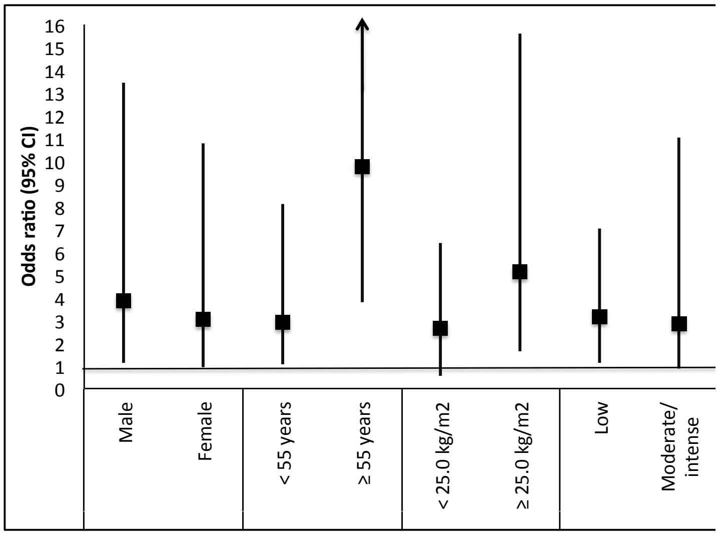

	OR	95% CI	*P*value
**Sex**[1]			
Male	3.85	1.10, 13.46	0.01
Female	3.06	0.91, 10.80	
**Age**[2]			
< 55 years	2.88	1.05, 8.08	0.001
≥ 55 years	9.77	3.78, 25.50	
**BMI**[3]			
< 25.0 kg/m2	2.66	0.53-6.42	0.13
≥ 25.0 kg/m2	5.10	1.66, 15.60	
**Physical activity**[4]			
Inactive	3.16	1.16, 7.02	0.02
Active/highly active	2.85	0.90, 11.01	
